# Direct insertion of the posterior cruciate ligament tibial attachment and its relationship with the medial meniscus: A histological study

**DOI:** 10.1002/jeo2.70563

**Published:** 2025-11-14

**Authors:** Akihiro Yamashita, Kosuke Tabuchi, Ryuki Hashida, Keishiro Kikuchi, Shotaro Kinouchi, Seiichi Inoue, Takashi Soejima, Akira Maeda, Shuji Horibe, Koichi Watanabe, Takahiro Okawa, Koji Hiraoka

**Affiliations:** ^1^ Department of Orthopedic Surgery Kurume University School of Medicine Kurume Fukuoka Japan; ^2^ Department of Orthopedic Surgery Kurume University Medical Center Kurume Fukuoka Japan; ^3^ Department of Sports Medicine and Science, Faculty of Human Health Kurume University Kurume Fukuoka Japan; ^4^ Hakata Knee & Sports Clinic Fukuoka Japan; ^5^ Seifu Hospital Orthopedics Sakai Osaka Japan; ^6^ Division of Gross and Clinical Anatomy, Department of Anatomy Kurume University School of Medicine Kurume Fukuoka Japan

**Keywords:** histology, medial meniscus, posterior cruciate ligament, PCL reconstruction, tibia

## Abstract

**Purpose:**

In this study, we aimed to identify the direct insertion of the tibial attachment of the posterior cruciate ligament (PCL) using histological examination and evaluate its anatomical relationship with the medial meniscus (MM).

**Methods:**

Twenty‐one formalin‐fixed cadaveric knees were examined. The PCL tibial attachment was analysed in sagittal (slices 1, 2 and 3) and coronal (slices 4, 5, 6 and 7) sections. Hematoxylin and eosin and Masson's trichrome staining were used for tissue evaluation. The tibial insertion length and direct insertion length were measured using ImageJ software. The relationship between direct insertion and anatomical parameters was analysed using Spearman's correlation coefficient.

**Results:**

The mean medial anteroposterior length was 52.1 ± 3.7 mm. The mean tibial insertion lengths of the PCL were 9.8 ± 1.7 mm (slice 1), 10.8 ± 1.1 mm (slice 2) and 8.5 ± 0.9 mm (slice 3). The mean direct insertion lengths were 8.5 ± 1.4 mm (slice 1), 9.7 ± 1.0 mm (slice 2) and 7.5 ± 0.9 mm (slice 3). Direct insertion in slice 2 was significantly correlated with medial anteroposterior length (*p* < 0.05). In the sagittal plane, the MM posterior root was adjacent to the anterior edge of the PCL tibial insertion, with a clear bony change‐point at the border. In the coronal plane, the medial tibial plateau and PCL were separated by the MM tibial attachment (slices 5 and 6).

**Conclusions:**

The tibial attachment area of the PCL was shorter than that reported in previous gross anatomical studies. These findings provide important anatomical insights for PCL reconstruction and are essential for preventing iatrogenic MM injuries during tibial tunnel creation.

**Level of Evidence:**

N/A.

**Abbreviations**
ACL: anterior cruciate ligament
AHLM: anterior horn of the lateral meniscus
H&E: hematoxylin and eosin
MAP: medial anteroposterior
ML: mediolateral
MM: medial meniscus
MRI: magnetic resonance imaging
PCL: posterior cruciate ligament

## INTRODUCTION

The posterior cruciate ligament (PCL) is an important structure that controls the posterior movement of the tibia [1, 5]. The PCL is a fibrous structure that connects the anterior part of the intercondylar notch of the medial femoral condyle to the posterior edge of the tibial attachment site. The posterior edge, known as the champagne glass drop‐off, spans the entire width of the tibial plateau [2, 21, 24, 27].

PCL injuries commonly result from sports activities, traffic accidents and falls [22]. When knee instability occurs, arthroscopic ligament reconstruction is the preferred treatment, aiming to restore anatomical structure and function. Achieving this goal requires detailed anatomical knowledge of the PCL [3, 6, 17, 26].

Several previous anatomical studies have reported actual measurements of the PCL tibial attachment [2, 8, 9, 18]. However, these investigations were limited to macroscopic anatomy, without histological confirmation of the attachment site. Recent technical notes and anatomical studies have described anatomical PCL reconstruction, reproducing both bundles and identifying tibial footprints in greater detail [7, 17]. Nevertheless, a systematic review of tibial tunnel placement in PCL reconstruction highlighted the lack of reliable anatomical landmarks for tunnel creation [19]. Histological evaluation of the ligament [20], bony landmarks [12] and the anatomical relationship between ligaments and the meniscus [15] is considered essential in ACL reconstruction. In contrast, detailed histological and anatomical information regarding the PCL tibial attachment and its relationship with the medial meniscus (MM) remains insufficient. Additionally, Laprade et al. [16] reported the risk of iatrogenic injury to the posterior root of the MM during tibial tunnel preparation for PCL reconstruction. To improve the accuracy of anatomical PCL reconstruction, it is necessary to identify the direct insertion at the PCL attachment site and clarify its anatomical relationship with the MM.

Therefore, in this study, we aimed to determine the position and length of the direct insertion of the PCL tibial attachment using histological examination and evaluate its anatomical relationship with the MM. It was hypothesised that the tibial attachment of the PCL possesses a histologically defined direct insertion and that it is closely adjacent to the posterior root of the MM.

## MATERIALS AND METHODS

This study was approved by the Ethics Committee of our university (approval number: [21101]). All procedures complied with local and international ethical guidelines and laws pertaining to the use of human cadaveric donors in anatomical research [14].

A total of 21 formalin‐fixed cadaveric knees (1 pair and 19 unpaired; 7 female and 13 male donors) were included in this study. The mean age of the donors at the time of death was 77.8 years (59–97 years). Knees were selected based on the presence of degenerative changes but with intact ligaments. The exclusion criteria were knees with marked deformity (equivalent to Kellgren–Lawrence grades 3 and 4), ligamentous damage, or confirmed tears in the posterior root of the MM.

All dissections were performed by a board‐certified orthopaedic surgeon with extensive experience in musculoskeletal anatomy. After carefully removing all the muscles and joint capsule surrounding the knee, the ACL, patellar tendon and collateral ligaments were released. The PCL was transected at the mid‐substance level, and using a bone saw, the proximal tibia was cut distal to the so‐called champagne‐glass drop‐off, which is a sharp depression site at the posterior margin of the tibia [2, 6]. The soft tissue around the PCL and MM was preserved to assess its position relative to the PCL.

**Figure 1:**
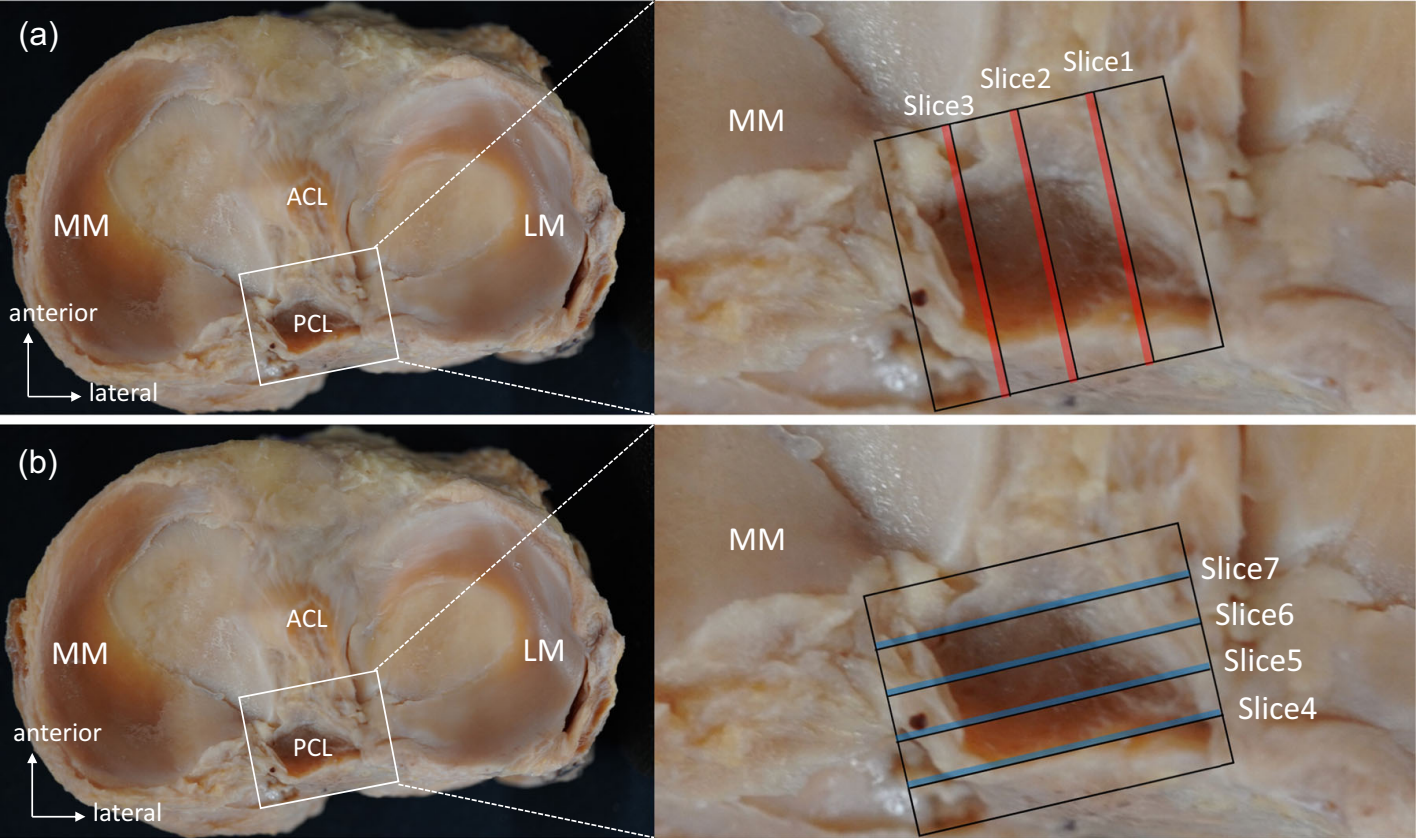
Sagittal and coronal sections of the PCL for histological evaluation. (a) Sagittal plane perpendicular to the champagne glass drop‐off. (b) Coronal plane parallel to the champagne‐glass drop‐off. ACL, anterior cruciate ligament; LM, lateral meniscus; MM, medial meniscus; PCL, posterior cruciate ligament.

Block specimens (approximately 25 × 25 × 20 mm) encompassing the PCL tibial attachment were prepared. Samples were delipidated using a xylene–methyl alcohol solution (1:1 v/v) for 24 h, followed by demineralisation for 5–10 days using a Plankyclo demineralisation solution. The duration of demineralisation was adjusted based on bone quality. Dehydration was accomplished using a graded alcohol series. For sagittal plane evaluation, the full width of the PCL was divided into three sections perpendicular to the champagne‐glass drop‐off using 15 knees, yielding four blocks of equal thickness. These split planes were designated as slices 1, 2 and 3 from the outside and used for histological evaluation (Figure 1a).

For coronal plane evaluation, the PCL was divided into four sections parallel to the champagne‐glass drop‐off using six knees, producing five blocks of equal thickness. These planes were designated as slices 4, 5, 6 and 7 from posterior to anterior and used for histological evaluation (Figure 1b). Prepared tissue sections were embedded in paraffin using an automated embedding machine, sliced at 5 µm thickness and stained with hematoxylin and eosin (H&E) and Masson's trichrome.

For macroscopic evaluation, the mediolateral (ML) width and medial anteroposterior (MAP) length of the tibial articular surface were measured in the superior view.

### Histological evaluation of the PCL tibial attachment

**Figure 2:**
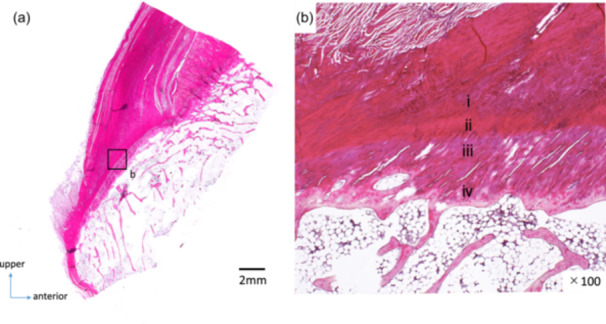
Histological findings at the posterior cruciate ligament tibial attachment site using hematoxylin and eosin staining. (a) Overall view. (b) ×100. (i) Ligament, (ii) noncalcified fibrocartilage layer, (iii) calcified fibrocartilage layer, (iv) bone.

Fifteen knees were divided in the sagittal plane. The position and attachment form of the PCL tibial attachment were observed in sagittal slices 1–3. The definition of the area showing the four‐layer structure of ligament–noncalcified fibrocartilage layer–calcified fibrocartilage layer–bone was based on the findings of previous studies on ligament and tendon attachment sites [4, 23]. This area was defined as the direct insertion site (Figure 2).

The PCL tibial insertion was defined as the region from the anterior margin of the attachment to the champagne‐glass drop‐off. Tibial insertion length and direct insertion length were measured and compared across slices. In slice 2, the relationship between tibial and direct insertion and variables such as age, sex, MAP length and tibial ML width was also analysed.

The length of each tissue was measured using ImageJ software (version 1.52k, National Institutes of Health). All histological staining was independently evaluated by a board‐certified orthopaedic surgeon (the author). Interobserver reliability was analysed using intraclass correlation coefficients (ICCs). Another board‐certified orthopaedic surgeon independently repeated all measurements to determine interobserver reliability, yielding an ICC (2.1) of 0.85.

### Anatomical relationship between the PCL tibial attachment and MM

In addition to 15 knees divided in the sagittal plane, 6 knees divided in the coronal plane were used to investigate the anatomical relationship between the PCL and MM. This relationship was evaluated in sagittal plane slices 1–3 and coronal plane slices 4–7.

### Statistical analysis

The correlation between the direct insertion length in slice 2 (central section) and age, sex, MAP length and ML width of the tibia was evaluated using Spearman's rank correlation coefficient. The normality of variables was assessed using the Shapiro–Wilk test. Most values were approximately normally distributed and are expressed as mean ± standard deviation. Differences in tibial and direct insertion lengths across sagittal slices were statistically analysed using analysis of variance, followed by post hoc testing with Tukey's test. JMP Pro 18 (JMP Statistical Discovery LLC, a SAS company) was used for statistical analysis. Statistical significance was set at *p* < 0.05.

## RESULTS

A total of 21 knees (7 females, 13 males; mean age 77.8 years, range 59–97 years) were analysed.

### Gross and histological evaluation of the PCL tibial attachment

The mean MAP length and ML width of the tibia were 52.1 ± 3.7 and 77.4 ± 5.7 mm, respectively. The posterior margin of the PCL tibial attachment coincided with the champagne glass drop‐off of the posterior tibial margin. Direct insertion was observed in all cases. The lengths of the PCL direct and tibial insertions for each slice are listed in Table 1. The tibial insertion lengths in slices 1 and 2 were significantly greater than in slice 3, but no significant difference was observed between slices 1 and 2. For direct insertion, slice 2 was significantly longer than slices 1 and 3, while the difference between slices 1 and 3 was not significant (Table 1).

**Table 1:** Lengths of the PCL tibial and direct insertions.

	Slice 1	Slice 2	Slice 3
Direct insertion (mm)	8.5 ± 1.4^a^ (5.6−10.8)	9.7 ± 1.0^b^ (7.8−11.4)	7.5 ± 0.9 (5.1−8.8)
Tibial insertion (mm)	9.8 ± 1.7^d^ (6.9−11.7)	10.8 ± 1.1^c^ (9.4−12.7)	8.5 ± 0.9 (7.0−8.8)

*Note*: The numbers in the table represent the means, standard deviations and ranges (minimum–maximum).

Abbreviation: PCL, posterior cruciate ligament.

a Slice 1 versus slice 2: There was a significant difference in direct insertion between the two groups.

b Slice 2 versus slice 3: There was a significant difference in direct insertion between the two groups.

^c^ Slice 2 versus slice 3: There was a significant difference in tibial insertion between the two groups.

^d^ Slice 1 versus slice 3: There was a significant difference in tibial insertion between the two groups.

### Correlation between direct insertion and anatomical parameters

Direct insertion length in slice 2 (central section) was significantly correlated with tibial MAP length (*ρ* = 0.5769; *p* = 0.0244). No significant correlations were found with ML width, age, or sex (ML: *ρ* = 0.3989, *p* = 0.1408; age: *ρ* = −0.0859, *p* = 0.7607; sex: *ρ* = 0.3780, *p* = 0.1648) (Table 2).

**Table 2:** Correlation between direct insertions in the central section of the PCL tibial attachment and investigated factors.

	MAP length of the tibia		ML width of the tibia		Age		Sex	
	*ρ*	*p*	*ρ*	*p*	*ρ*	*p*	*ρ*	*p*
Direct insertion	0.5769	0.0244^a^	0.3989	0.1408	−0.0859	0.7607	0.3780	0.1648

Abbreviations: MAP, medial anteroposterior; ML, mediolateral; PCL, posterior cruciate ligament.

a Significant difference.

### Anatomical relationship between the PCL tibial attachment and MM

**Figure 3:**
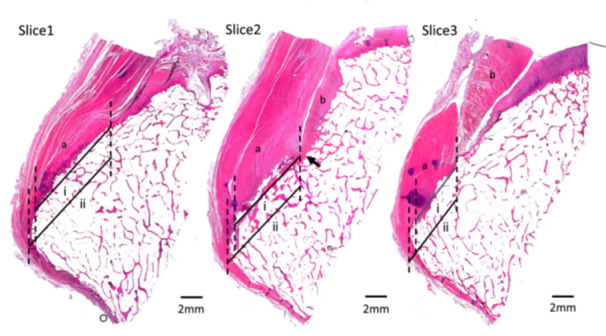
Anatomical relationship between the posterior cruciate ligament tibial attachment and the medial meniscus in the sagittal plane. The arrow indicates the point of the bony change. (a) Posterior cruciate ligament. (b) Posterior root of the medial meniscus. (ⅰ) Direct insertion. (ⅱ) Tibial insertion.

In sagittal slices 2 and 3, the MM was adjacent to the PCL tibial attachment. In slice 2, the MM was located at the posterior root, and a distinct bony change‐point was identified at its boundary with the PCL tibial attachment (Figure 3). In the coronal plane, the MM tibial attachment was positioned between the medial tibial plateau (MTP) and the PCL (slices 5 and 6).

**Figure 4:**
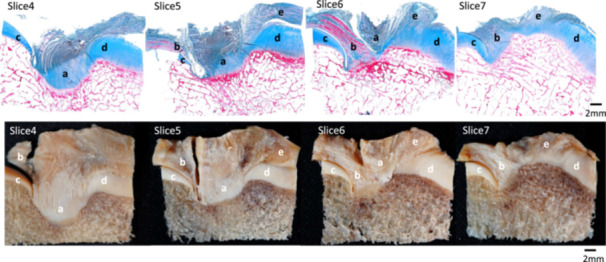
Anatomical relationship between the posterior cruciate ligament tibial attachment and the medial meniscus in the coronal plane. (a) Posterior cruciate ligament. (b) Posterior root of the medial meniscus. (c) Medial tibial plateaus. (d) Lateral tibial plateaus. (e) Lateral meniscus.

In contrast, in slices 4, 5 and 6, the lateral tibial plateau was directly adjacent to the PCL without intervening structures (Figure 4).

## DISCUSSION

The most important finding of this study is that the direct insertion of the PCL measured 8.5 ± 1.4 mm (slice 1), 9.7 ± 1.0 mm (slice 2) and 7.5 ± 0.9 mm (slice 3), while the tibial insertion measured 9.8 ± 1.7 mm (slice 1), 10.8 ± 1.1 mm (slice 2) and 8.5 ± 0.9 mm (slice 3). These values are shorter than those reported in previous macroscopic studies [9, 25]. Additionally, in slice 2, the MM was in the region of the posterior root, and a bony change point was identified at its boundary with the PCL tibial attachment. To our knowledge, this is the first study to provide a detailed histological evaluation of the PCL tibial attachment and its boundary with the MM.

Few gross anatomical studies have been conducted on the PCL tibial attachment length. Girgis et al. [9] reported the mean PCL tibial attachment length of 13.4 mm and width of 13 mm, while Van Dommelen et al. [25] obtained comparable values. In contrast, the PCL insertion lengths obtained in our study were shorter than those previously reported for PCL tibial attachments based on gross anatomy. There are several possible explanations for this observation. First, racial differences may play a role, as our specimens were exclusively Asian, whereas earlier studies examined European and US populations. Second, the measurement techniques used in this study differed from those used in previous reports. Iwanaga et al. reported that histological observations are more accurate than gross measurements, supporting the validity of our findings [13]. Taken together, our results suggest that the histological PCL tibial attachment area is smaller than that reported in gross anatomical studies.

Additionally, the PCL tibial footprint correlated with sex, height and tibial plateau dimensions in studies using magnetic resonance imaging (MRI) [10, 11]. In our data, direct insertion of the PCL correlated with the tibial MAP length but not with age, sex, or tibial ML width. In future studies, we plan to apply regression analyses to provide a more robust evaluation. For this purpose, we estimated the required sample size using G*Power (version 3.1.9.7). Assuming an effect size of *f*² = 0.15 (medium), *α* = 0.05, power = 0.80, and two predictors, the required sample size is 55. Thus, future studies with an appropriately powered cohort are warranted to investigate the relationship between direct insertion of the PCL and tibial MAP length.

In this study, the MM posterior root was adjacent to the anterior edge of the PCL tibial insertion, and a clear bony change‐point was identified at the border in the sagittal plane. Kusano et al. [15] elucidated the anatomical locations of the ACL and lateral meniscus attachments. The ACL attachment was found to be an L‐shape, and the two were separated by a distinct bony boundary. They reported that the anterior horn of the lateral meniscus (AHLM) is a useful marker for creating bone tunnels and that knowing the exact location of the ACL and AHLM can reduce the risk of lateral meniscal injury during ACL reconstruction. Similarly, the positional relationship between the PCL and MM was investigated in this study. In slice 2 of the sagittal section, the anterior portion of the PCL tibial attachment was separated from the posterior root of the MM by a bone change point. When the PCL tunnel is placed anterior to the attachment area, iatrogenic MM injury can occur. Furthermore, the MM was positioned between the PCL and the MTP in slice 5 of the coronal section. Consequently, even if the PL tunnel is medial to the attachment area, MM injury can occur.

LaPrade et al. reported that a champagne‐glass drop‐off can serve as a useful landmark for creating a PCL tunnel, thus minimising the risk of iatrogenic MM injury during PCL reconstruction [2]. The results of this study support those of LaPrade et al.

In summary, because the MM is adjacent to the PCL anteriorly and medially, a tibial tunnel should be created so that it does not deviate anteriorly or medially due to the risk of MM injury. Understanding the exact positional relationship between the PCL and MM may similarly reduce the risk of MM injury during PCL reconstruction.

### Limitations

This study has some limitations. First, we utilised only cadaver knees and did not perform imaging evaluations using computed tomography or MRI. Second, the study was conducted only on Asians; therefore, it was not possible to observe racial differences. Third, the average age of the patients was high because of the use of cadaveric knees, and the results may differ from those of younger patients because of ligament degeneration. Increased age is associated with histological and anatomical variations in ligaments. Although specimens were selected based on the absence of degenerative changes, the potential influence of age on the histological findings cannot be excluded.

Finally, all specimens were formalin‐fixed, which can cause tissue shrinkage and stiffening, potentially reducing measured distances and altering soft tissue elasticity. These changes may distort spatial relationships and bias absolute measurements compared with fresh or in vivo tissue. Therefore, the quantitative values obtained in this study should be interpreted with caution.

### Conclusions

This study provided a detailed histological analysis of the PCL tibial attachment. The PCL was found to be adjacent to the MM posterior root, with a distinct bony change‐point marking their boundary. Understanding the exact positional relationship between the PCL and MM may help reduce the risk of MM injury during PCL reconstruction.

## AUTHOR CONTRIBUTIONS

Akihiro Yamashita, Kosuke Tabuchi, Takashi Soejima, Akira Maeda, Shuji Horibe and Koji Hiraoka participated in the conception and design of the study. Akihiro Yamashita, Keishiro Kikuchi, Shotaro Kinouchi and Seiichi Inoue acquired the data. Ryuki Hashida conducted the statistical analyses. Koichi Watanabe and Shuji Horibe provided support with expert anatomical knowledge. Akihiro Yamashita wrote the manuscript. Ryuki Hashida, Keishiro Kikuchi and Kosuke Tabuchi edited the first draft. Takahiro Okawa and Koji Hiraoka assisted with editing the manuscript. All authors read and approved the final manuscript.

## CONFLICT OF INTEREST STATEMENT

The authors declare no conflicts of interest.

## ETHICS STATEMENT

This study was approved by the Institutional Review Board of Kurume University (approval number: 21101).

## Data Availability

The datasets generated and/or analysed in the current study are available from the Harvard Dataverse repository: https://doi.org/10.7910/DVN/5TSCGG.
